# Targeted Overexpression of Mitochondrial ALDH2 in Coronary Endothelial Cells Mitigates HFpEF in a Diabetic Mouse Model

**DOI:** 10.3390/biom15071029

**Published:** 2025-07-16

**Authors:** Guodong Pan, Bipradas Roy, Emmanuel Oppong Yeboah, Thomas Lanigan, Roland Hilgarth, Rajarajan A. Thandavarayan, Michael C. Petriello, Shailendra Giri, Suresh Selvaraj Palaniyandi

**Affiliations:** 1 Division of Hypertension and Vascular Research, Department of Internal Medicine, Henry Ford Health System, Detroit, MI 48202, USA; gpan1@hfhs.org (G.P.); bipradas.roy@duke.edu (B.R.); ho6538@wayne.edu (E.O.Y.); 2 Department of Physiology, Wayne State University, Detroit, MI 48202, USA; 3 Vector Core, Biomedical Research Core Facilities, University of Michigan Medical School, Ann Arbor, MI 48109, USA; lanigant@med.umich.edu (T.L.); rhilgar@med.umich.edu (R.H.); 4 Department of Cardiovascular Sciences, Houston Methodist Research Institute, Houston, TX 77030, USA; 5 Institute of Environmental Health Sciences, Wayne State University, Detroit, MI 48202, USA; michael.petriello@wayne.edu; 6 Department of Pharmacology, Wayne State University, Detroit, MI 48202, USA; 7 Department of Neurology, Henry Ford Health, Detroit, MI 48202, USA; sgiri1@hfhs.org

**Keywords:** 4-hydroxy-2-nonenal (4HNE), aldehyde dehydrogenase 2 (ALDH2), heart failure with preserved ejection fraction (HFpEF), coronary vascular endothelial cells (CVECs), gene transfer, type 2 diabetes mellitus (T2DM), adeno-associated virus serotype 9 expressing vascular endothelial (VE)-cadherin

## Abstract

Heart failure (HF) has become an epidemic, with a prevalence of ~7 million cases in the USA. Despite accounting for nearly 50% of all HF cases, heart failure with a preserved ejection fraction (HFpEF) remains challenging to treat. Common pathophysiological mechanisms in HFpEF include oxidative stress, microvascular dysfunction, and chronic unresolved inflammation. Our lab focuses on oxidative stress-mediated cellular dysfunction, particularly the toxic effects of lipid peroxidation products like 4-hydroxy-2-nonenal (4HNE). Aldehyde dehydrogenase 2 (ALDH2), a mitochondrial enzyme, plays a vital role in detoxifying 4HNE and thereby protecting the heart against pathological stress. ALDH2 activity is reduced in various metabolic stress-mediated cardiac pathologies. The dysfunction of coronary vascular endothelial cells (CVECs) is critical in initiating HFpEF development. Thus, we hypothesized that ectopic overexpression of ALDH2 in CVECs could mitigate metabolic stress-induced HFpEF pathogenesis. In this study, we tested the efficacy of intracardiac injections of the *ALDH2* gene into CVECs in *db*/*db* mice—a model of obesity-induced type 2 diabetes mellitus (T2DM)—and their controls, *db*/*m* mice, by injection with ALDH2 constructs (AAV9-VE-cadherin-hALDH2-HA tag-P2A) or control constructs (AAV9-VE-cadherin-HA tag-P2A-eGFP). We found that intracardiac ALDH2 gene transfer increased ALDH2 levels specifically in CVECs compared to other myocardial cells. Additionally, we observed increased ALDH2 levels and activity, along with decreased 4HNE adducts, in the hearts of mice receiving *ALDH2* gene transfer compared to control GFP transfer. Furthermore, *ALDH2* gene transfer to CVECs improved diastolic function compared to GFP control alone. In conclusion, ectopic ALDH2 expression in CVECs can contribute, at least partially, to the amelioration of HFpEF.

## 1. Introduction

Heart failure (HF) affects 6.7 million Americans, a number expected to rise to 8.5 million by 2030 [1]. The lifetime risk of developing HF is approximately 24%, meaning one in four adults will develop HF [1]. Despite HF with a preserved ejection fraction (HFpEF) accounting for roughly 50% of all HF cases, it remains challenging to treat and study due to its heterogeneous etiopathology and comorbidities. Several chronic metabolic conditions, including obesity, type 2 diabetes mellitus (T2DM), hypertension, metabolic syndrome, kidney disease, pulmonary disease, and atrial fibrillation are associated with HFpEF [2,3]. For instance, epidemiological data indicate that a significant portion of HFpEF patients are overweight or obese [4], emphasizing the strong link between adiposity and diastolic dysfunction. T2DM is also highly prevalent in HFpEF [5] and promotes myocardial stiffness through insulin resistance, chronic inflammation, and endothelial dysfunction. Given the alarming rise in all these metabolic diseases, we chose to focus on metabolic stress-associated HFpEF.

The hallmarks of obesity and type 2 diabetes mellitus (T2DM), such as dyslipidemia and insulin resistance, can in themselves lead to cardiac pathophysiology. Lipotoxicity, characterized by the accumulation of lipids in non-adipose tissues such as the myocardium, causes cellular dysfunction and apoptosis and contributes to adverse cardiac remodeling [6]. Insulin resistance disrupts myocardial substrate utilization, leading to impaired energy efficiency and cardiac performance [7]. Metainflammation arises from adipose tissue dysfunction and exacerbates endothelial injury and myocardial fibrosis [6,8]. Oxidative stress is another important causal mechanism in HFpEF, arising from an imbalance between reactive oxygen species (ROS) and antioxidant defenses. The resulting secondary effects cause widespread damage to myocardial cells and structures, thereby aggravating HFpEF pathology [9]. Additionally, mitochondrial dysfunction plays a critical role by impairing ATP production and increasing oxidative burden, which further compromises cardiac energetics.

Oxidative stress leads to aberrations in cellular homeostasis and function. Among the oxidative stress-mediated cellular damage mechanisms, we focus on ROS-mediated lipid peroxidation driven by 4-hydroxy-2-nonenal (4HNE) and similar reactive aldehydes as a culprit of cardiac complications [10]. Unlike ROS, 4HNE and other lipid peroxidation products can easily diffuse across membranes and covalently modify proteins and DNA in the cytoplasm, organelles, and nucleus, far from their site of origin [11]. We have shown increased 4HNE adducts in the cardiac tissue of T2DM mice compared to control mice [12,13,14]. Additionally, the activity of aldehyde dehydrogenase 2 (ALDH2), a primary enzyme that oxidizes cellular aldehydes into carboxylic acids, is reduced in the hearts of T2DM mice [12,13,14,15]. This reduction in ALDH2 activity exacerbates aldehyde accumulation, contributing to oxidative damage and cardiac dysfunction [13]. ALDH2 serves as a central regulator of redox homeostasis during oxidative stress [16]. ALDH2 has the highest affinity for 4HNE (Km = 0.9 μM) among ALDH isozymes, making it the most effective enzyme for 4HNE detoxification under physiological conditions [17,18]. The cardioprotective function of ALDH2 has been demonstrated in multiple in vivo and ex vivo models of myocardial ischemia–reperfusion injury [19,20]. The importance of ALDH2 in cardiovascular diseases is also supported by studies in ALDH2 knockout (KO) mouse models, which display increased susceptibility to cardiac injury, enhanced oxidative stress, and reduced aldehyde clearance [21]. These findings imply ALDH2’s critical role in protecting the heart under metabolic and oxidative stress conditions. Moreover, a common single point mutation in the ALDH2 gene (E487K), known as ALDH2*2, dramatically reduces the enzyme’s catalytic efficiency to only 3–30% of wild-type activity [19,22]. This mutation, present in approximately 700 million East Asians, is associated with increased susceptibility to myocardial infarction and diabetic complications [17]. ALDH2*2 mutant mice with T2DM exhibit significantly worse cardiac function compared to their non-mutant counterparts, further highlighting the clinical relevance of ALDH2 dysfunction in diabetic cardiomyopathy [12,13].

The cardiac component of HFpEF is increasingly recognized as being closely linked to coronary microvascular dysfunction [23]. This process is believed to begin with inflammation, dysfunction, apoptosis, and the eventual rarefaction of coronary vascular endothelial cells (CVECs). Microvascular rarefaction—defined as a reduction in myocardial capillary density—has been consistently observed in HFpEF patients, as shown in histopathological studies [24]. For instance, Mohammed et al. (2015) reported significantly decreased micro vessel density in myocardial tissue of HFpEF patients, correlating inversely with the extent of myocardial fibrosis [24]. This microvascular compromise leads to impaired myocardial perfusion. A key consequence is the reduction in coronary flow reserve (CFR), which reflects the inability of coronary vessels to meet increased oxygen demands during stress. Paulus and Tschöpe proposed a paradigm in which systemic inflammation—driven by comorbidities like hypertension, obesity, and diabetes—leads to endothelial inflammation and oxidative stress [25]. This in turn reduces nitric oxide (NO) bioavailability, impairs vasodilation, and promotes myocardial stiffening and fibrosis.

In addition to inflammation and rarefaction, impaired angiogenesis contributes to the pathology. The inadequate formation of new micro vessels compromises the heart’s adaptive capacity to ischemia, leading to chronic oxygen supply–demand mismatch. This imbalance promotes cardiomyocyte hypoxia, which activates fibrotic signaling pathways and contributes to myocardial fibrosis—central to the development of diastolic dysfunction in HFpEF [13,26]. Collectively, metabolic stressors contribute to cellular damage which all underpins the heterogeneous and progressive nature of HFpEF. The clinical phenotypes of HFpEF vary based on the severity and diversity of these comorbidities and the affected organ systems and cell types. However, replicating all of these comorbidities in a single preclinical animal model is challenging [3]. However, the increasing prevalence of obesity and T2DM, as well as their culmination in HFpEF, led us to use leptin receptor-deficient *db*/*db* mice as our preclinical model for this study, as these mice develop obesity by 4 weeks of age, T2DM by 12 weeks, and HFpEF by 24 weeks [14].

Taken together, these findings highlight that coronary microvascular dysfunction—driven by endothelial cell inflammation, rarefaction, reduced CFR, and impaired angiogenesis—plays a central role in the development of myocardial fibrosis and diastolic dysfunction, culminating in HFpEF. Therapeutic strategies aimed at preserving microvascular integrity and restoring endothelial function may hold promise in mitigating the progression of this complex syndrome. We demonstrated that 4HNE contributes to CVEC apoptosis, inflammation, rarefaction, and dysfunction, which is aggravated by low ALDH2 activity in vitro, ex vivo, and in vivo studies [27,28,29]. The pro-inflammatory activation of CVECs occurs slowly as they encounter metabolic stress from the blood in T2DM, metabolic syndrome, and obesity [30,31,32]. This facilitates the attachment and transmigration of circulating activated inflammatory cells like monocytes to CVECs, leading to cardiac remodeling processes such as inflammation, hypertrophy, and fibrosis. These changes result in a stiff myocardium, causing diastolic dysfunction with preserved systolic function [9,30]. The search for new therapies for HFpEF has been hampered by a limited understanding of its complex pathogenesis. Despite the diversity of comorbidities, they converge on oxidative stress, microvascular dysfunction, and chronic inflammation as causal factors in HFpEF pathogenesis and progression. In this study, we planned to focus on oxidative stress-induced 4HNE-mediated dysfunction of CVECs as a critical initial step of HFpEF development in metabolic diseases. Decreasing cellular 4HNE will reduce CVEC damage. Our goal was to determine whether enhancing aldehyde detoxification in CVECs through targeted ALDH2 overexpression improves cardiac function in a preclinical model of HFpEF driven by obesity-induced T2DM. We hypothesize that metabolic stress increases 4HNE accumulation in CVECs, leading to oxidative injury and diastolic dysfunction, which can be mitigated by targeted ALDH2 overexpression.

## 2. Materials and Methods

### 2.1. Animals: db/db Mouse

We employed the *db*/*db* mouse, a leptin receptor mutant mouse, exhibiting elevated plasma insulin levels at 10–14 days, elevated blood glucose levels at 4–8 weeks, and developing cardiac dysfunction around 16–20 weeks of age. We bred *db*/*db* mice and their age-matched controls, *db*/*m*, with a C57BL/6 background, in our facility. The mice were bred and grown in our animal care facility and genotyped by Transnetyx Inc. (Cordova, TN, USA). We used *n* = 6 mice per group based on sample size power analysis and previous experience. We compared the results between the *db*/*db* mice and their controls *db*/*m* mice. We included all data. The animal protocols were approved by the Wayne State University Institutional Animal Care and Use Committee.

### 2.2. Intracardiac Delivery of ALDH2 Gene Using AAV9 Viral Vector

**Figure 1:**
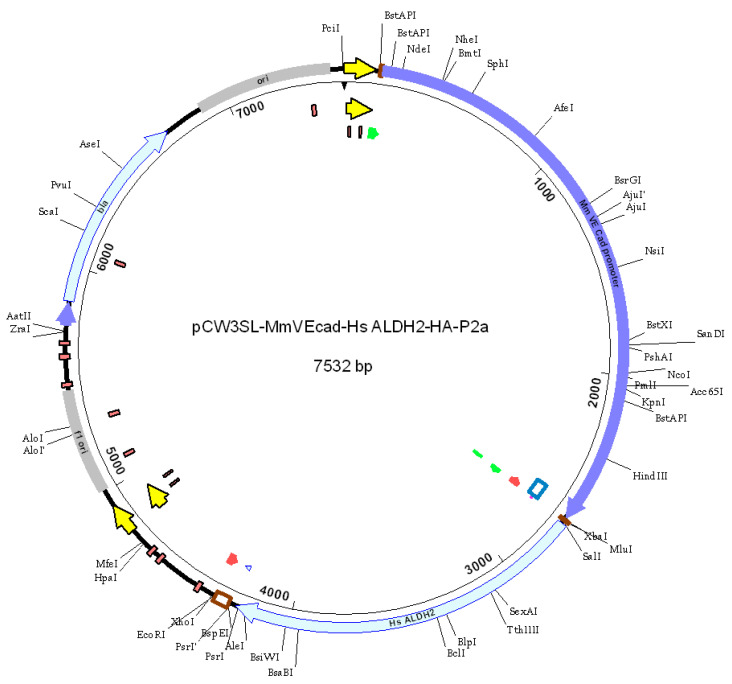
Map of ALDH2 construct. Human ALDH2 was subcloned into the pCW3SL AAV vector. The ALHD2 HA P2a was PCR amplified from a previously generated construct, pCWB cTNT ALDH2 HA P2a eGFP using the following primers (SalI 5′-TCTAGACGCGTCGACCACCATGTTGCGCGCTGCCGCCCGCT, EcorI R 5′-AGGTTGATTATCTCGAGCGGAATTCCTAAGGACCGGGGTTTTCTTCCACGTCTC. The VE-cadherin promoter was amplified from mouse genomic DNA. Promoter and transgene were inserted into the BstAPI/EcoRI digested pCW3SL vector. Map shows single cutting restriction enzyme sites.

Mice were randomly selected to receive either the ALDH2 gene or GFP, based on their assigned cages. After being anesthetized with 3% isoflurane, they were positioned supine under a heating lamp. We felt the beating heart by touching with fingers and then injected it through intact skin. A syringe with a 29-G needle was inserted and 10 μL/site of adeno-associated viral vector 9 (AAV9) (10^12^ viral particles, diluted in PBS) containing either the ALDH2 gene (AAV9-VE-cadherin-hALDH2-HA tag-P2A) or eGFP - (AAV9-VE-cadherin-HA tag-P2A eGFP) asshown in Figure 1 for a construct map) were injected into 4 places in the left ventricle from the apex to base. After injection, the mice were kept on a heating pad until they regained consciousness, then returned to the animal facilities for monitoring. After 3 weeks, the mice were sacrificed to assess cardiac ALDH2 levels, activity, and 4HNE protein adducts.

### 2.3. Cardiac Ultrasound

To determine whether CVEC-targeted ALDH2 overexpression preserves cardiac function in the setting of metabolic stress, we performed transthoracic echocardiography using the Vevo 3100 micro-ultrasound system (VisualSonics, Toronto, ON, Canada). Mice were anesthetized with 3–5% isoflurane for induction and maintained under 1–2% via inhalation throughout imaging. All acquisitions were analyzed using Vevo LAB software to ensure consistency across experimental groups. A heart rate (HR) of approximately 600 bpm was maintained during echocardiography to reflect the physiological range observed in conscious adult mice (typically 600–700 bpm). Maintaining this rate minimizes anesthesia-induced bradycardia and ensures accurate assessment of diastolic parameters. Lower heart rates can distort Doppler waveforms, specifically by fusing E and A waves, compromising the interpretation of filling dynamics. Additionally, prior studies have shown that measurements are more reproducible and stable when the heart rate is maintained above 475 bpm during imaging.

We assessed both systolic and diastolic function in AAV9-GFP- and AAV9-ALDH2-treated *db*/*m* and *db*/*db* mice. Systolic function was evaluated by measuring the ejection fraction (%EF) and left ventricular dimensions—end-systolic diameter (LVESD) and end-diastolic diameter (LVEDD)—to capture the global contractile performance.

To assess structural remodeling, we measured the thickness of the anterior (LVAW;d) and posterior (LVPW;d) walls during diastole using M-mode imaging. Increased wall thickness indicates concentric hypertrophy, a common feature of pressure overload and diastolic dysfunction in HFpEF. We then focused on detailed characterization of the diastolic performance. The E/A ratio, obtained by pulse wave Doppler (PWD), reflects the balance between early passive filling and late atrial contraction—shifts in this ratio indicate impaired relaxation. Isovolumic relaxation time (IVRT), also measured via PWD, quantifies the interval between aortic valve closure and mitral valve opening; prolonged IVRT signals delayed myocardial relaxation. Finally, we assessed the E/e′ ratio using tissue Doppler imaging, which provides a noninvasive estimate of left ventricular filling pressure and correlates with increased diastolic stiffness and left atrial pressure.

By combining structural and functional parameters, this approach enabled us to characterize the HFpEF phenotype in our model and to determine whether ALDH2 overexpression in CVECs preserves myocardial diastolic performance under conditions of obesity-driven type 2 diabetes mediated metabolic stress.

### 2.4. Immunofluorescence Staining

Frozen cardiac tissue sections were used for immunostaining to assess transfection efficiency by examining eGFP fluorescence in mice that received AAV9-ALDH2 and AAV9-GFP. Additionally, staining for CD31 and ALDH2 was performed using an CD31 (PECAM-1) monoclonal antibody (Thermo Fisher Scientific Inc., Bothell, WA, USA, 14-0311-82; 1:100) and an anti-ALDH2 mouse monoclonal antibody (Thermo Fisher Scientific Inc., MA5-17029; 1:100) with overnight incubation at 4 °C. Secondary antibodies were conjugated with Alexa Fluor 546 (Invitrogen, Pittsburgh, Pennsylvania, USA, A11030, excitation at 556 nm, emission at 573 nm) at a concentration of 1:500 and incubated at room temperature for 1 h. Immunofluorescence-positive staining was analyzed using a fluorescent microscope (Olympus IX8, Waltham, MA, USA) and an image analyzer (Olympus IX2-UCB, Waltham, MA, USA). Representative micrographs were selected and presented.

### 2.5. Western Immunoblotting Using Proteins from Isolated Cardiac Cells and Tissue

In brief, *db*/*db* hearts were digested with collagenase to generate a single-cell suspension. CVECs were isolated from this suspension using magnetic beads conjugated to anti-CD31 antibodies, enriching for the CD31^+^ population. The remaining CD31^−^ fraction was passed through a 40 µm cell strainer to remove larger cardiomyocytes. The final CD31^−^ cell fraction with <40 µm primarily consisted of non-endothelial, non-myocyte cells, including cardiac fibroblasts and macrophages.

Each of the isolated cell types as well as the heart tissue were separately homogenized using T-PER™ Tissue Protein Extraction Reagent (78510; Thermo Fisher Scientific Inc. Bothell, WA, USA), and then proteins were isolated and quantified.

Next, Western blotting was performed as described earlier [13] using the protein samples: The samples were separated on SDS–polyacrylamide gels by electrophoresis before being transferred to Immobilon-P membranes. Protein levels were determined by probing using the specific antibodies mentioned below: anti-CD 31 mouse monoclonal antibody (CD31 (PECAM-1) Monoclonal Antibody (390)) (14-0311-81; Invitrogen, Pittsburgh, Pennsylvania, USA, 1:1000), anti-CD68 (KP1) monoclonal antibody (14-0688-82; eBioscience, Thermo Fisher Scientific Inc. Bothell, WA, USA, 1:1000), Alpha-smooth muscle actin mouse monoclonal antibody (CGA7) (sc-53015; Santa Cruz Biotechnology Inc., Santacruz, CA, USA, 1:3000), Cardiac Troponin T Monoclonal Antibody (13-11) (MA5-12960; Thermo Fisher Scientific Inc., Bothell, WA, USA, 1:2000), and GAPDH mouse monoclonal antibody (G-9) (sc-365062; Santa Cruz Biotechnology Inc. Santacruz, CA, USA, 1:3000). The bound antibodies were visualized with horseradish peroxidase (HRP)-coupled secondary antibodies. After obtaining the cell marker blots, the membrane was stripped and re-probed for GAPDH as a loading control.

The protein samples from the heart tissue homogenates were separated, run in the gel, transferred to the membrane as mentioned above, and probed with anti-ALDH2 mouse monoclonal antibody (MA5-17029; Thermo Fisher Scientific Inc.; Bothell, WA, USA, 1:1000) and anti-4HNE-Cys/His/Lys rabbit polyclonal antibody (393207-M; Millipore Sigma, Burlington, MA, USA, 1: 1000). After these two exposures, the same membrane was stripped and then probed with anti-β actin mouse monoclonal antibody (sc-47778; Santacruz Biotechnology, Santacruz, CA, USA, 1:3000) as a loading control. Finally, the band intensities were quantified using ImageJ 1.54p software and presented as arbitrary units.

### 2.6. ALDH Activity Assay

ALDH2 activity was measured following the method described previously elsewhere [15,19,33]. After homogenizing whole heart tissue, ALDH2 enzymatic activity in the cardiac tissue homogenates was determined spectrophotometrically by monitoring the reduction of NAD+ to NADH at 340 nm. All assays were conducted at 25 °C in a 0.1 M sodium pyrophosphate buffer (pH 9.5) with 2.4 mM NAD+ as a cofactor and 10 mM acetaldehyde as the substrate.

### 2.7. Statistical Analysis

Data are presented as the mean ± standard error of the mean (S.E.M). The *db*/*m* mice with AAV-GFP and AAV-ALDH2 were compared with *db*/*db* mice with AAV-GFP and AAV-ALDH2. We used one-way ANOVA for group multiple comparisons, and post hoc analysis was performed using Student’s *t*-test. We used Microsoft Excel for all statistical analysis.

## 3. Results

### 3.1. Effective and Selective Transduction of Injected Genes into CVECs via Intracardiac Transfer

**Figure 2:**
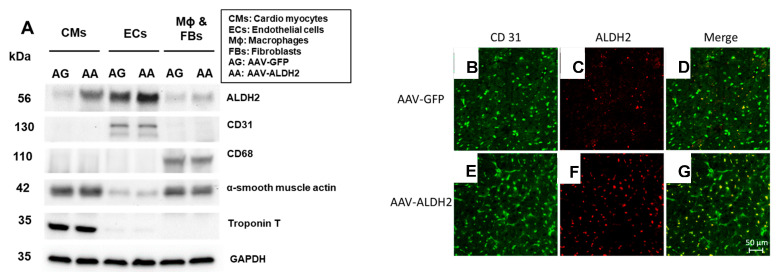
Overexpression of ALDH2 specifically in CVECs by the AAV-9 viral vector with a VEC-specific promoter by directly injecting into the *db*/*db* mouse hearts. Among the isolated myocardial cells, ALDH2 was overexpressed only in coronary ECs, not Mϕs, FBs, and CMs as shown in the immunoblots with their specific markers such as CD31, CD68, α-smooth muscle actin, and troponin T, respectively, and the loading control, GAPDH (**A**). Representative micrographs (**B**–**G**) from hearts of AAV-GFP– and AAV-ALDH2–treated mice demonstrate immunopositivity for CD31 (green fluorescence) and ALDH2 (red fluorescence). The micro graphical images of immunostaining show colocalization of endothelial marker CD31 with ALDH2 signal (**D**,**G**), supporting successful transgene expression in coronary endothelial cells.. Original western blots can be found at Supplementary Materials.

Intramyocardial injections of VE Cadherin containing the AAV9 vector show that CD31^+^ CVECs overexpress ALDH2 protein but other cardiac cells do not, such as cardiac troponin T-containing cardiomyocytes, CD68^+^ macrophages, and alpha-smooth muscle actin-containing myofibroblasts (Figure 2A) in *db*/*db* hearts. The CD31 immunopositivity in both AAV9-GFP and AAV-9 ALDH2 was shown by green florescence (Figure 2B,E). ALDH2 transduction indicated with enhanced red fluorescence was observed in the AAV9-ALDH2 groups with immunostaining (Figure 2F) compared to AAV9-GFP (Figure 2C). The colocalization of both green and red indicates the increased ALDH2 expression in CD31^+^ ECs (Figure 2D compared to Figure 2G).

### 3.2. Intracardiac Injection of ALDH2 Gene with EC-Specific VE Cadherin Promoter Increased the Myocardial ALDH2 Level and ALDH2 Activity and Reduced 4HNE Adducts in db/db and db/m Hearts

**Figure 3:**
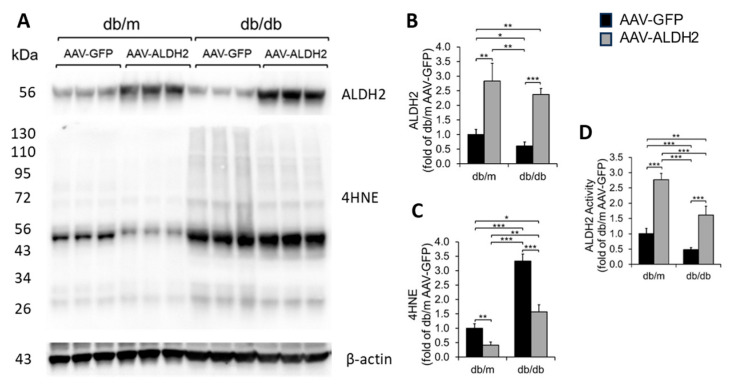
ALDH2 and 4HNE levels and ALDH2 activity in the myocardium with ALDH2 overexpression specifically in CVECs. Immunoblot images of ALDH2, 4HNE adducts, and β-actin (**A**). The quantification data of levels of ALDH2 (**B**) and 4HNE adducts (**C**), as well as ALDH2 activity (**D**), are shown. The data are the mean ± SEM. *n* > 3. * *p* < 0.05; ** *p* < 0.01; *** *p* < 0.001. Original western blots can be found at Supplementary Materials.

We found cardiac ALDH2 protein levels (Figure 3A,B) were significantly increased in both *db*/*m* and *db*/*db* mice that received an intracardiac injection of the ALDH2 gene with the EC-specific VE Cadherin promoter compared to their counterparts that received an intracardiac injection of the GFP gene with the EC-specific VE Cadherin promoter gene. We also found that the intracardiac transfer of the ALDH2 gene reduced myocardial 4HNE protein adducts in *db*/*m* and *db*/*db* mice compared to their counterparts that received the GFP gene (Figure 3A,C). Finally, we found that intracardiac transfer of the ALDH2 gene increased myocardial ALDH2 activity in *db*/*m* and *db*/*db* mice compared to their counterparts that received the GFP gene (Figure 3D).

### 3.3. Specific Overexpression of the ALDH2 Gene in CVECs Improved Diastolic Dysfunction in db/db Mice

**Figure 4:**
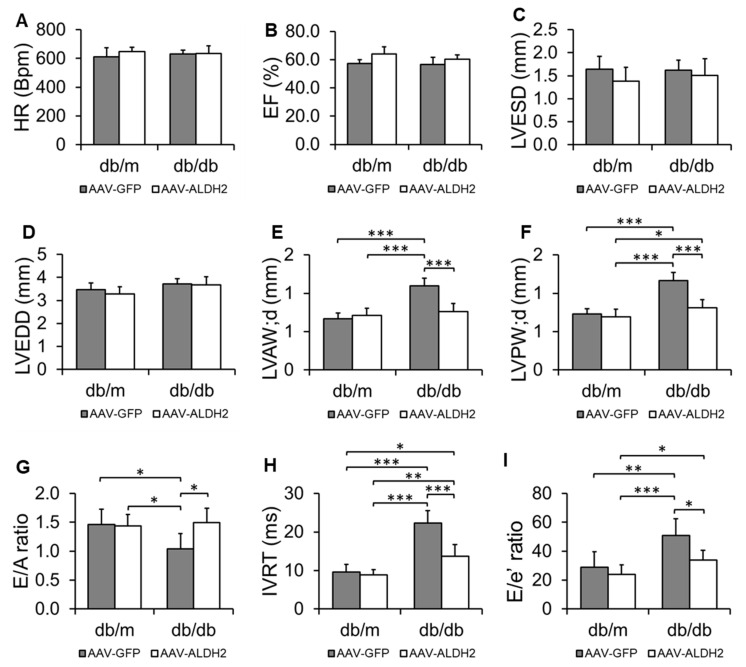
Changes in cardiac functional parameters. Echocardiographic evaluation of cardiac function in *db*/*m*, *db*/*db*, AL, and AF mice. The heart rate (HR) (**A**), percentage ejection fraction (%EF) (**B**), left ventricular end-systolic diameter (LVESD) (**C**), and left ventricular end-diastolic diameter (LVEDD) (**D**) showed no significant differences among groups. Left ventricular anterior wall thickness in diastole (LVAW;d) (**E**) and posterior wall thickness in diastole (LVPW;d) (**F**) were measured. The E/A ratio, defined as the ratio of early (**E**) to late (**A**) diastolic peak mitral inflow velocities, was evaluated (**G**). Isovolumic relaxation time (IVRT) (**H**) and the E/e′ ratio, calculated by dividing the peak early mitral inflow velocity (**E**) by the early diastolic mitral annular velocity (e′) (**I**) were evaluated. AAV9-GFP or AAV9-ALDH2 was administered to *db*/*m* and *db*/*db* mice as indicated. The data are the mean ± SEM. *n* = 6. * *p* < 0.05; ** *p* < 0.01; *** *p* < 0.001.

HR, % EF, LVESD, and LVEDD were unchanged across all groups, indicating preserved systolic function (Figure 4A–D). In *db*/*m* controls, the overexpression of ALDH2 in CVECs had no impact on LVAW;d, LVPW;d, the E/A ratio, IVRT, or E/e′, confirming no off-target effects in healthy mice (Figure 4E–I). As expected, *db*/*db* mice treated with AAV9-GFP exhibited signs of diastolic dysfunction relative to *db*/*m* controls: LVAW;d and LVPW;d were significantly increased (Figure 4E,F), while the E/A ratio was reduced and IVRT and E/e′ were elevated (Figure 4G–I), consistent with impaired relaxation and increased filling pressure. Importantly, ALDH2 overexpression in *db*/*db* mice significantly attenuated these changes. Compared to AAV9-GFP-treated *db*/*db* mice, AAV9-ALDH2- treated *db*/*db* mice showed reductions in LVAW;d and LVPW;d (Figure 4E,F), an improved E/A ratio (Figure 4G), shortened IVRT (Figure 4H), and lowered E/e′ (Figure 4I), indicating partial restoration of diastolic function.

## 4. Discussion

We found that intracardiac ALDH2 gene transfer profoundly increased ALDH2 levels in CVECs compared to other myocardial cell types, achieving precise cellular targeting that is critical for mechanistic insight and potential therapeutic translation. This was confirmed through immunostaining that demonstrated robust ALDH2 expression in CD31^+^ endothelial cells but not in cardiomyocytes, fibroblasts, or macrophages, thereby excluding off-target effects that could confound interpretation. Correspondingly, the hearts of mice receiving an ALDH2 gene transfer exhibited elevated ALDH2 protein levels and enzymatic activity with a concurrent reduction in 4HNE adducts. This molecular signature of reduced 4HNE adducts in the myocardial microenvironment translated into improved diastolic function in the treated group relative to mice receiving green fluorescent protein (GFP) transfer, without affecting systolic function, as expected for the HFpEF model.

Our results underscore a critical concept: targeted enhancement of cellular aldehyde detoxification pathways within the coronary endothelium may restore endothelial health, thereby addressing fundamental pathological features of HFpEF, such as endothelial dysfunction, capillary rarefaction, and endothelial cell death. This is particularly important because HFpEF is increasingly recognized not just as a cardiomyocyte disease but as a syndrome driven by non-myocyte contributors including the coronary microvasculature [25]. CVECs are uniquely susceptible to metabolic and oxidative insults due to their high exposure to circulating metabolites and ROS [34,35]. Excessive ROS generation in the context of metabolic stress leads to lipid peroxidation, producing reactive aldehydes such as 4HNE that covalently modify proteins, disrupt signaling pathways, and ultimately impair endothelial cell function and survival [36]. By enhancing ALDH2 expression specifically in CVECs, we facilitate clearance of these toxic aldehydes, thus mitigating the cascade of oxidative injury that contributes to endothelial dysfunction and loss.

Obesity and diabetes are established clinical risk factors for HFpEF and profoundly exacerbate coronary microvascular pathology [37]. Obese patients demonstrate poorer exercise capacity and worse outcomes compared to non-obese individuals, an effect attributed in part to metabolic stress-induced endothelial damage and microvascular rarefaction [38]. Human studies have documented markedly reduced coronary microvascular density in individuals with a body mass index (BMI) >30, suggesting that excess adiposity directly impairs coronary endothelial integrity [39]. Animal models further validate these observations: Wistar–Kyoto rats and C57BL6/NCrSlc mice fed a high-fat diet develop significant cardiac capillary rarefaction and diastolic dysfunction [40]. A recent study using continuous infusion of angiotensin II and phenylephrine in mice recapitulated multiple HFpEF features including exercise intolerance, pulmonary edema, concentric hypertrophy, and importantly, microvascular rarefaction and fibrosis [41]. These findings not only replicate human pathology but also highlight the interplay between systemic hypertension, neurohormonal activation, and coronary endothelial injury.

The *db*/*db* mouse model used in our study combines obesity, T2DM, and HFpEF phenotypes, characterized by reduced coronary angiogenesis and capillary density [42]. Mechanistic analyses reveal that metabolic stress suppresses key pro-angiogenic signaling pathways such as the fibroblast growth factor-2 (FGF-2), early growth response protein 1 (EGR-1), and vascular endothelial growth factor A (VEGF-A) axis [43]. Dipeptidyl peptidase 4 (DPP4) has been identified as a causal mediator in this suppression, representing a link between metabolic dysregulation and impaired angiogenesis [44]. These molecular derangements create a hostile microenvironment that favors capillary rarefaction. Oxidative stress compounds this injury; excess ROS and lipid peroxidation products, particularly 4HNE, accumulate in coronary vessels and impair endothelial cell proliferation, migration, and new vessel formation [13,14,27,29]. For example, 4HNE adducts disrupt endothelial nitric oxide synthase (eNOS) function and inhibit VEGF-mediated angiogenic responses, thereby aggravating microvascular loss and impairing myocardial perfusion [45].

It is reported that the majority of the HFpEF patients have T2DM, and diabetic patients with end-stage HFrEF display significantly lower cardiac capillary density than their non-diabetic counterparts [46,47]. Consistent with this, diabetic transgenic pig models demonstrate capillary rarefaction which can be partially reversed by AAV-mediated VEGFA gene therapy, leading to reduced left ventricular fibrosis and end-diastolic pressure, despite an unchanged ejection fraction [48]. Together, these clinical and preclinical studies illuminate how metabolic and oxidative stresses synergistically promote endothelial loss and dysfunction, fueling myocardial remodeling and the diastolic dysfunction characteristic of HFpEF.

Recent insights suggest that improving CVEC health could interrupt this pathological cycle. Our approach utilized an AAV9 vector with a vascular endothelial cadherin (VEC)-specific promoter to selectively overexpress ALDH2 in coronary endothelial cells. AAV9 was chosen for its exceptional cardiac tropism and high transduction efficiency, as evidenced by studies showing > 200-fold myocardial transduction compared to other serotypes [49]. AAV vectors persist episomally in non-dividing cells, allowing sustained gene expression without chromosomal integration, which reduces the oncogenic risk [50]. Nonetheless, immune responses to AAV capsids remain a challenge, with 30–50% of humans harboring neutralizing antibodies that can limit efficacy [51]. Our strategy to target CVECs mitigates off-target effects and maximizes therapeutic benefit.

Functionally, ALDH2 detoxifies reactive aldehydes like 4HNE, preventing protein adduct formation that impairs endothelial signaling and survival. Our findings endorsed this notion that ALDH2 overexpression in CVECs elevated enzyme activity and decreased 4HNE adducts, directly attenuating oxidative injury at the cellular source. This biochemical improvement correlated with preserved systolic function but, more importantly, with significant enhancement of diastolic parameters including LVAW;d, LVPW;d, favorable E/A ratio shifts, shortened IVRT, and reduced E/e′ ratios. These combined improvements reflect enhanced myocardial relaxation and reduced filling pressures, which are pathognomonic of HFpEF progression.

In this context, the translational relevance of our findings is further underscored by clinical studies investigating endothelial-targeted therapies in HFpEF. For instance, a prospective pilot trial involving trans endocardial CD34^+^ cell transplantation in 30 HFpEF patients showed no improvement in diastolic function or exercise capacity after six months of medical therapy alone, but significant improvements following CD34^+^ cell therapy. Specifically, the E/e′ ratio decreased by ~32%, NT-proBNP dropped by ~36%, and the 6 min walk test (6MWT) distance increased by approximately 9%, suggesting enhanced diastolic function and exercise tolerance [52]. Similarly, in a study of 55 dilated cardiomyopathy patients randomized to CD34^+^ cell transplantation or control, the treated group experienced a significant increase in % EF and exercise capacity, as well as reduced NT-proBNP and mortality rates [53]. These data collectively support that therapies targeting the endothelial compartment can yield clinically meaningful benefits in cardiac function and patient outcomes.

Our earlier in vitro studies have established that 4HNE impairs CVEC angiogenesis by inhibiting proliferation and migration. Inhibition of ALDH2 worsened this effect, while ALDH2 activation restored angiogenic capacity [29]. Building on these mechanistic insights, the current in vivo study targeting CVEC-specific ALDH2 overexpression in an obesity–T2DM-driven HFpEF model demonstrates for the first time that directly modulating endothelial oxidative resilience translates into functional cardiac benefits. Prior systemic ALDH2 gene transfer studies demonstrated cardioprotection against ischemia/reperfusion injury by lowering 4HNE levels [54], but the current CVEC-focused strategy addresses the emerging paradigm that endothelial dysfunction and capillary rarefaction are central to HFpEF pathology.

The broader implications of these findings align with transcriptomic analyses from human HFpEF myocardium showing the downregulation of angiogenesis-related pathways, reinforcing that coronary rarefaction is not just a correlate but a contributor to disease progression [55]. Interventions that preserve or restore coronary endothelial health, as exemplified by ALDH2 gene therapy, offer promising avenues to arrest or reverse HFpEF progression.

Gene therapy has a transformative potential for cardiovascular diseases, offering possibilities for durable, one-time interventions that address underlying mechanisms rather than symptomatic management. However, significant challenges remain, including immune responses to vectors, off-target effects, cost, and regulatory hurdles. Precise targeting, as achieved here via endothelial-specific promoters and cardiac-tropic AAV9 vectors, reduces the risk of adverse events and maximizes therapeutic windows. Nonetheless, preexisting immunity to AAV capsids, particularly in humans carrying neutralizing antibodies, may limit efficacy and pose safety concerns [56,57]. Furthermore, recent clinical experience with high-dose AAV9 therapies revealed dose-dependent toxicities, such as liver injury [58] and neurotoxicity [59], necessitating rigorous patient screening, monitoring, and immunosuppression protocols.

Future Directions: This study demonstrates that CVEC-targeted ALDH2 overexpression reduces 4HNE accumulation and improves diastolic function in a preclinical model of HFpEF. To build on these findings, future studies will examine mitochondrial function, oxidative stress markers, inflammatory cytokines, and myocardial fibrosis to better define the downstream effects of aldehyde detoxification. Although AAV9 was delivered locally to the heart, comprehensive biodistribution and safety profiling across peripheral organs will be planned to confirm specificity and evaluate off-target effects.

In parallel, efforts to improve delivery precision and reduce immunogenicity will explore ligand-targeted nanoparticles, non-viral vectors, and engineered AAVs. Translating ALDH2-based therapies to clinical settings will ultimately require long-term studies incorporating coronary microvascular imaging, biomarkers of oxidative injury, and detailed assessments of cardiac structure and function in HFpEF.

Limitations of the Study: While our construct was designed to drive endothelial-specific ALDH2 expression using the VE-cadherin promoter, Figure 2A reveals a detectable ALDH2 signal in both CD31^+^ endothelial cells and cardiomyocytes, particularly in the AAV-ALDH2 group. This likely reflects a combination of local AAV9 tropism and limited promoter leakiness. AAV9 is known for its strong cardiomyocyte tropism, especially with intracardiac delivery, which can lead to unintended transduction of adjacent non-target cells despite promoter targeting. In parallel, although the VE-cadherin promoter is widely used to achieve endothelial-enriched expression, prior studies have reported context-dependent leakiness, with low-level activity observed in non-endothelial lineages under certain conditions or with truncated promoter fragments. Despite this, ALDH2 expression in CD31+ ECs was markedly more robust, and the dominant phenotypic improvements—restored capillary density, reduced coronary rarefaction, and improved diastolic performance—are consistent with endothelial rescue as the primary mechanism of benefit. Nonetheless, we acknowledge the possibility that ALDH2 expression in cardiomyocytes may contribute to the observed cardiac protection. Future studies using lineage-restricted reporters or conditional expression systems will be essential to further dissect cell-type-specific contributions to the therapeutic effects.

Although the E/A ratio is frequently used to assess diastolic function, its trajectory in *db*/*db* mice remains variable across studies. Some reports suggest a biphasic pattern—initially decreased due to impaired relaxation, followed by pseudonormalization as the left atrial pressure increases, and later a decline with worsening disease. In our earlier study [14], the E/A ratio remained reduced even at 6 months, consistent with earlier-stage dysfunction. Importantly, this reduction was accompanied by elevated E/e′ and prolonged IVRT, indicating impaired myocardial relaxation and increased filling pressure. These complementary indices strengthen the interpretation that our *db*/*db* mice exhibited diastolic dysfunction consistent with a pre-pseudonormalized or intermediate HFpEF stage.

## 5. Conclusions

In conclusion, our study demonstrates that selective ALDH2 overexpression in coronary vascular endothelial cells substantially reduces oxidative stress as evidenced by decreased 4HNE adducts and enhances diastolic cardiac function without impairing systolic performance. These findings suggest that coronary endothelium can be a therapeutic target in HFpEF and highlight the potential of CVEC-specific metabolic modulation to improve myocardial resilience and function. As the field moves towards precision therapies addressing the endothelial–metabolic axis, ALDH2 gene therapy in CVECs offers a promising, translationally relevant strategy to counteract HFpEF progression, particularly in metabolically compromised patients burdened by obesity and diabetes.

## Data Availability

All the data are within the manuscript. All materials were obtained from commercial vendors.

## Supplementary Materials

The following supporting information can be downloaded at: https://www.mdpi.com/article/10.3390/biom15071029/s1, Original Images for Blots.
