# Supplementary figures and images for: Targeted Overexpression of Mitochondrial ALDH2 in Coronary Endothelial Cells Mitigates HFpEF in a Diabetic Mouse Model
Source: Biomolecules. 2025 Jul 16;15(7):1029. doi: 10.3390/biom15071029 (PMC12293769; doi:10.3390/biom15071029)

Figure 2-raw data

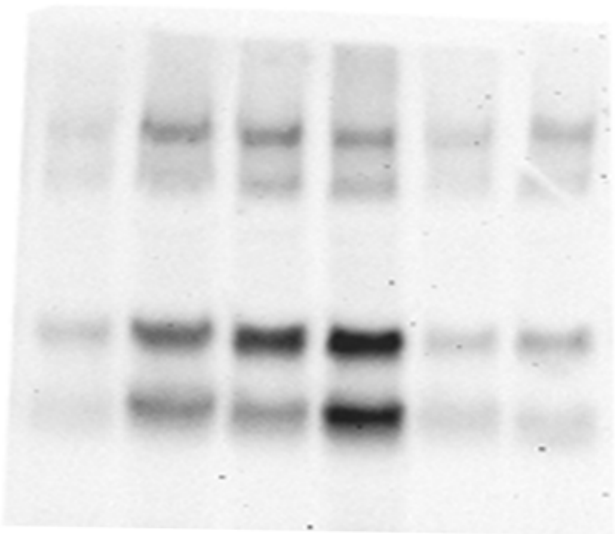

ALDH2

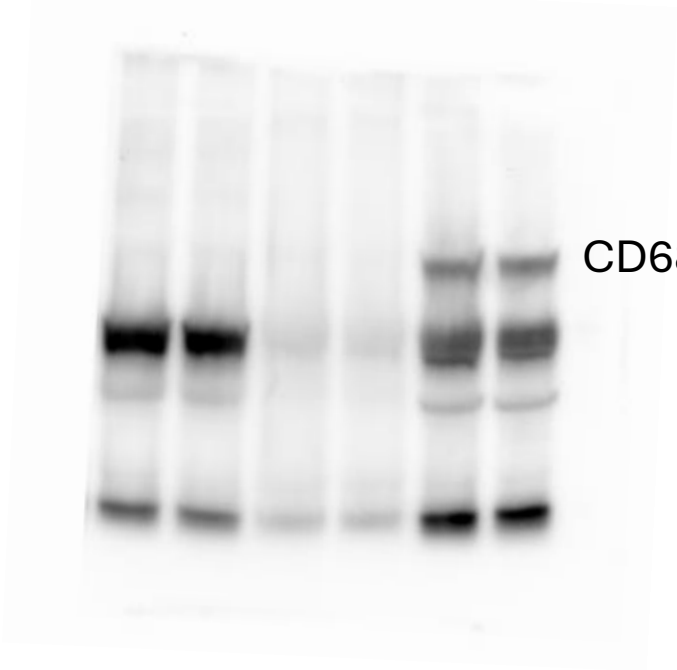

CD68

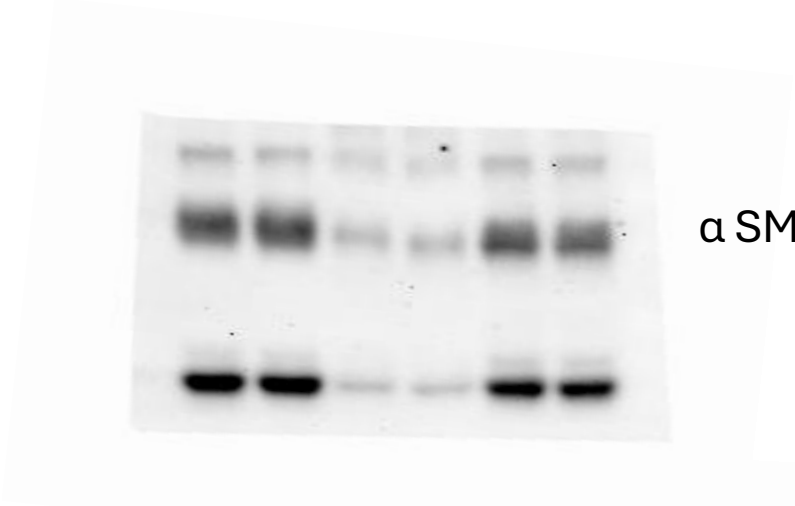

$\alpha$  SM

GAPDH

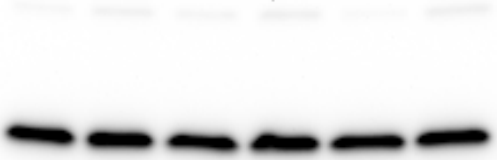

TNT

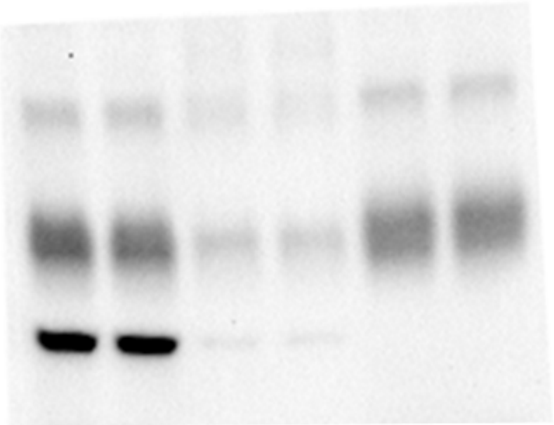

CD31

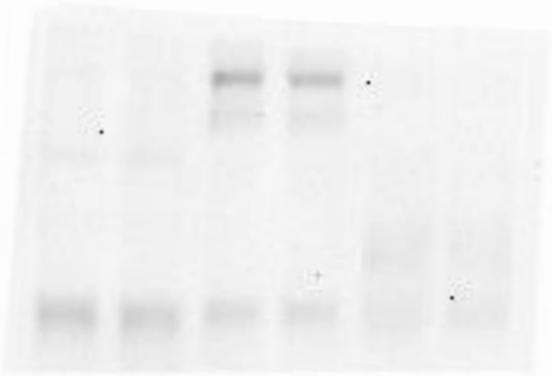

Figure 3-raw data

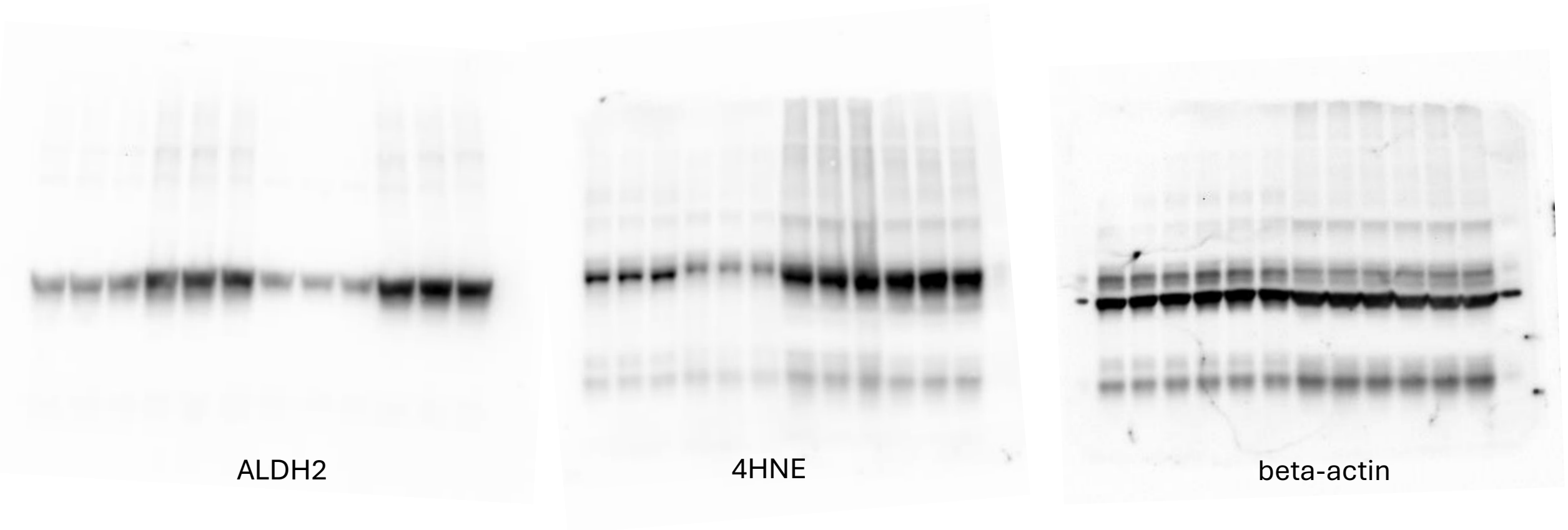

Supplement: Supplementary file 1 [file biomolecules-15-01029-s001.zip › biomolecules-3685476-supplementary.pdf]
